# Heparin-bound chemokine CXCL8 monomer and dimer are impaired for CXCR1 and CXCR2 activation: implications for gradients and neutrophil trafficking

**DOI:** 10.1098/rsob.170168

**Published:** 2017-11-08

**Authors:** Prem Raj B. Joseph, Kirti V. Sawant, Krishna Rajarathnam

**Affiliations:** 1 Department of Biochemistry and Molecular Biology, University of Texas Medical Branch, Galveston, TX, USA; 2 Sealy Center for Structural Biology and Molecular Biophysics, University of Texas Medical Branch, Galveston, TX, USA; 3 Department of Microbiology and Immunology, University of Texas Medical Branch, Galveston, TX, USA

**Keywords:** chemokine, glycosaminoglycan, GPCR, gradients, neutrophil, NMR

## Abstract

Chemokine CXCL8 plays a pivotal role in host immune response by recruiting neutrophils to the infection site. CXCL8 exists as monomers and dimers, and mediates recruitment by interacting with glycosaminoglycans (GAGs) and activating CXCR1 and CXCR2 receptors. How CXCL8 monomer and dimer interactions with both receptors and GAGs mediate trafficking is poorly understood. In particular, both haptotactic (mediated by GAG-bound chemokine) and chemotactic (mediated by soluble chemokine) gradients have been implicated, and whether it is the free or the GAG-bound CXCL8 monomer and/or dimer that activates the receptor remains unknown. Using solution NMR spectroscopy, we have now characterized the binding of heparin-bound CXCL8 monomer and dimer to CXCR1 and CXCR2 receptor N-domains. Our data provide compelling evidence that heparin-bound monomers and dimers are unable to bind either of the receptors. Cellular assays also indicate that heparin-bound CXCL8 is impaired for receptor activity. Considering dimer binds GAGs with higher affinity, dimers will exist predominantly in the GAG-bound form and the monomer in the free form. We conclude that GAG interactions determine the levels of free CXCL8, and that it is the free, and not GAG-bound, CXCL8 that activates the receptors and mediates recruitment of blood neutrophils to the infected tissue.

## 1. Introduction

A hallmark of infection is the immediate and robust recruitment of circulating neutrophils to the target tissue [1–4]. Chemokines mediate trafficking of neutrophils and other cell types to distal and remote locations in various tissues and organs [5–8]. Humans express around 50 chemokines, and all share a similar structural fold, exist as monomers and dimers (and some as higher order oligomers), and exert their function by binding G-protein-coupled receptors and sulfated glycosaminoglycans (GAGs) [9–13]. A subset of seven chemokines characterized by the highly conserved N-terminal ELR motif recruit neutrophils by activating CXCR1 and CXCR2 receptors [14,15]. Neutrophil-activating chemokines (NACs), released at the site of infection by resident cells, form concentration gradients that serve as beacons and guide the blood neutrophils to the infected site. Functional studies for NAC CXCL1 and CXCL8 have established that both monomers and dimers function as high-affinity CXCR2 agonists and that the CXCL8 monomer alone functions as a high-affinity CXCR1 agonist [16–20].

GAGs, such as heparan sulfate (HS) and chondroitin sulfate (CS), are linear sulfated polysaccharides ubiquitously expressed on many cell types. They are present on endothelial and epithelial cells covalently attached to core proteins and are the glycan part of proteoglycans (PGs) [21–23]. PGs are also an integral part of the extracellular matrix, and exist as non-covalent macromolecular complexes with proteins such as collagen and laminin [24]. GAG interactions determine the makeup of gradients, which dictate the flux and duration of neutrophil egress [25–34]. PG ectodomains can also be cleaved by proteases such as matrix metalloproteases. Therefore, chemokine binding to GAGs of PG ectodomains can also regulate neutrophil trafficking and has been shown to be essential for successful resolution of inflammation [35].

Neutrophil recruitment is dependent on the local CXCL8 concentration that can vary by many orders of magnitude as a function of time and space. Further, it must be remembered that all four species, chemokine monomers and dimers in the free and the GAG-bound forms, will exist as they are always in equilibrium [36–38]. Solution NMR studies for various NACs have shown that the dimer, compared to the monomer, binds GAG with much higher affinity [39–42].

Directed movement of leucocytes has been historically attributed to soluble chemotactic gradients [43–46]. However, on the basis of electron microscopy observations that CXCL8 was immobilized on tissue GAGs, it was proposed that solid-phase haptotactic and not chemotactic gradients mediate recruitment [30]. It was also argued that soluble gradients are unlikely to exist under flow conditions [29]. More recent intravital imaging studies have also been interpreted to indicate that GAG-bound chemokine is presented to the receptors on leucocytes [6,26]. Most importantly, at this time, there is no direct experimental evidence for a chemokine·GAG·receptor ternary complex and that GAG-bound chemokine can activate the receptors on leucocytes.

Structures of the CXCL8 monomer and dimer are known, and their receptor and GAG interactions have been well characterized [40,47–53]. In this study, we specifically ask whether the heparin-bound CXCL8 monomer or dimer can bind either the CXCR1 or CXCR2 receptor. HS has a modular structure with variable sulfated sequences (defined as NS domain) separated by sequences lacking all or most of these modifications (defined as NAc domain), and a transition region defined as a mixed NAc/NS domain [22]. CXCL8 and most other chemokines preferentially bind to the NS domains. Heparin is more uniformly sulfated and therefore functions as a surrogate for HS NS domains. Characterization of ternary complexes first requires characterizing the binary complexes of the CXCL8 monomers and dimers bound to heparin, CXCR1 and CXCR2. The receptor N-domain functions as a critical ligand-docking site, and previous studies have shown that the isolated receptor N-domain peptides can be used to capture N-domain interactions outside the context of the intact receptor [17,51,53–59]. Our previous studies using CXCL8 wild-type (WT) and monomer and dimer constructs have also shown that the monomer is the high-affinity CXCR1 ligand and that the dimer binds CXCR1 with much lower affinity [51,53,55]. We have also characterized CXCL8 binding to heparin oligosaccharides and found that the dimer, compared to the monomer, binds heparin with higher affinity [40].

Our current studies unambiguously indicate that the heparin-bound monomer and dimer are unable to bind either CXCR1 or CXCR2, that only the free chemokine can bind the receptor, and that heparin-bound chemokine is impaired for receptor activity. We conclude that GAG interactions determine the levels of free CXCL8, the free and not GAG-bound CXCL8 that mediates receptor activation, and that chemotactic gradients play a prominent role in mediating neutrophil trafficking.

## 2. Material and methods

### 2.1. Cloning, expression and purification

CXCL8 WT, CXCL8 trapped dimer and V27P/E29P monomer mutant (hereafter referred to as CXCL8 monomer), and the CXCR1 N-domain 29mer peptide (R1) were recombinantly expressed as a thioredoxin fusion protein with His-tag in *Escherichia coli* BL21(DE3) strain and purified as described previously [53,60]. Monomer design involved mutating dimer interface residues V27 and E29 to proline. We have shown previously that this double proline CXCL8 (V27P/E29P) mutant is monomeric and is as active as the WT monomer in functional assays [60]. Synthetic CXCR2 N-domain 43mer peptide (R2) was purchased from Aapptec (KY, USA) [18].

^15^N-labelled CXCL8 variants were produced by growing cells in minimal medium containing ^15^NH_4_Cl as the nitrogen source. Transformed cells were grown to an *A*_600_ ∼ 0.6, and induced with 1 mM isopropyl *β*-d-thiogalactopyranoside overnight at 23°C. The fusion protein was purified using a nickel-NTA column, and treated with Factor Xa that resulted in precise cleavage of the protein with no extraneous amino acids from thioredoxin or the His-tag. The cleaved protein was purified using reverse-phase high-performance liquid chromatography column. The purity and molecular weight of the proteins were confirmed using matrix-assisted laser desorption/ionization mass spectrometry.

### 2.2. NMR spectroscopy

^15^N-labelled proteins were prepared in 50 mM sodium phosphate pH 7.0 buffer containing 1 mM DSS (2,2-dimethyl-2-silapentanesulfonic acid), 1 mM sodium azide and 10% ^2^H_2_O (v/v). ^1^H-^15^N heteronuclear single-quantum coherence (HSQC) spectra were acquired at 30°C on a Bruker Avance III 800 MHz (with a TXI cryoprobe) or 600 MHz (with a QCI cryoprobe) spectrometers. Spectra were processed with NMRPipe [61] and analysed using NMRView [62] or Bruker Topspin 3.2 software.

The starting protein concentrations for the different HSQC titrations experiments were between 50 and 100 µM. At these concentrations, WT CXCL8 exists predominantly as a dimer and the V27P/E29P mutant as a monomer. The heparin octasaccharide (dp8) and heparin 14mer (dp14) were purchased from Iduron (Manchester, UK). Aliquots from a stock solution of dp8 (10 mM) or dp14 (5 mM) were added to the protein samples, and a series of ^1^H-^15^N HSQC spectra were collected until essentially no changes in chemical shifts were observed. The final CXCL8 : heparin molar ratios for the monomer and dimer were approximately 1 : 10. Aliquots of a stock solution (2 mM) of R1 or R2 were added to the heparin-bound CXCL8 until no changes in chemical shift perturbation were observed. The final protein : peptide molar ratios for these titrations were approximately 1 : 10. A similar excess of receptor peptides was added for titrations to heparin-bound monomer or dimer.

### 2.3. Analytical ultracentrifugation

Sedimentation velocity experiments were performed for CXCL8, CXCL8·dp8 complex and a mixture of CXCL8, R1 and dp8 using a Beckman Coulter XL-A analytical ultracentrifuge. The samples were prepared in 50 mM sodium phosphate buffer, pH 7.0, containing 100 mM NaCl. UV absorbance at 280 nm for the different samples was between 0.3 and 0.9. A 400 µl aliquot of the buffer and sample was loaded into the reference and sample compartments of a double-sector cell, assembled with 1.2 cm charcoal-Epon centerpiece and quartz windows. Sedimentation experiments were performed at 50 000 r.p.m. and 25°C. A total of 400 scans were collected. The datasets were analysed using SEDFIT software (v. 9.4) with the *c*(*s*) continuous size distribution model, allowing the frictional ratio to float [63].

### 2.4. Neutrophil receptor activity

Whole blood was obtained from healthy non-smoking individuals with donor consent under a human subject study protocol approved by the institutional review board at the University of Texas Medical Branch at Galveston. Neutrophils (greater than 85% pure) were purified as described previously [64]. A total of 2 × 10^5^ neutrophils in HBSS were plated in a flat-bottomed black microplate and kept at room temperature for 1 h. The cells were then loaded with FLIPR calcium assay 6 dye for 2 h. 100 nM of CXCL8 variants was mixed with different concentrations of heparin (Iduron, Manchester, UK) and immediately added to dye-loaded cells. The changes in fluorescence were monitored (*λ*_ex_ 485 nm, *λ*_em_ 525 nm) every 5 s for 240–500 s at room temperature using a Flexstation III microplatereader (Molecular Devices). Heparin by itself did not induce any Ca^2+^ release. The agonist response was determined by expressing the maximum change in fluorescence in arbitrary units over baseline. Statistical significance was determined using ANOVA followed by Tukey's post hoc analysis; *p < 0.05, **p < 0.01, ***p < 0.001.

## 3. Results

NMR chemical shifts are highly sensitive to their environment and are excellent probes for detecting binding-induced local structural changes. NMR is also ideal for characterizing weak binding interactions that are not easily accessible by other biophysical techniques. In a ^1^H–^15^N HSQC spectrum, each cross peak corresponds to the amide resonance of a specific residue, and binding-induced local/global changes can be measured from a series of HSQC titration experiments. In this study, we used binding-induced chemical shift changes as structural probes to characterize whether CXCR1 or CXCR2 binding to heparin-bound CXCL8 monomers or dimers results in a ternary complex.

For our current studies, we used the following CXCL8 and receptor constructs. For dimer interactions, we used the WT CXCL8 that exists predominantly as a dimer at the concentrations used for the NMR experiments and will be referred to as the CXCL8 dimer. For monomer interactions, we used the V27P/E29P mutant, which is monomeric at the concentrations used in the NMR studies, and is as active as the WT in both *in vitro* and animal model studies [60]. For functional studies, we used the disulfide trapped dimer [17]. For CXCR1 interactions, we used a 29mer peptide that has been characterized for binding to the CXCL8 monomer and dimer [53]; for CXCR2 interactions, we used a 43mer peptide that has been previously used for binding to the CXCL1 monomer and dimer [18]. We chose heparin dp8 on the basis of our previous studies that showed dp8 optimally spans the binding surface on the CXCL8 monomer and dimer and also provided good-quality NMR spectra [40]. In addition, we also used a longer heparin dp14 for some of the titrations.

### 3.1. Binding of CXCR1 to heparin-bound CXCL8 monomer

**Figure 1.**
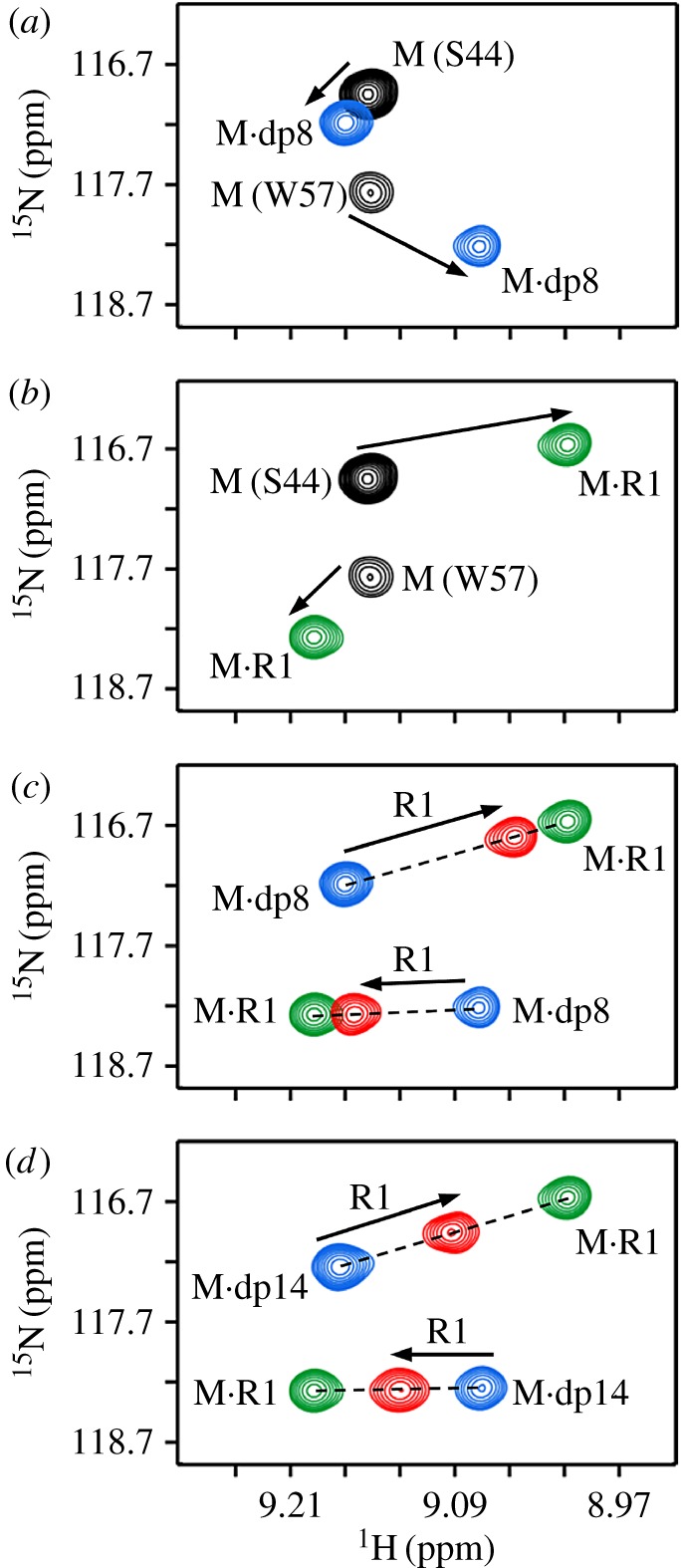
Binding of the CXCR1 N-domain to heparin-bound monomer. Section of the ^1^H–^15^N HSQC spectrum showing heparin dp8 (*a*) and R1 (*b*) binding to the CXCL8 monomer. The free (M) and final (M·dp8 and M·R1) cross-peaks for residues S44 and W57 are shown in black, blue and green, respectively. (*c*,*d*) Sections of the same region on adding R1 to M·dp8 and M·dp14 complexes. On successive addition of R1, the new cross-peaks fall on a straight line joining the M·R1 and M·dp8 peaks. The final peak positions are shown in red.

We first describe NMR characteristics of the CXCR1 N-domain (R1) and heparin dp8 binding to the CXCL8 monomer (M). Titration profiles for residues S44 and W57 are shown in figure 1. On titrating heparin, we observed significant chemical shift changes for a subset of residues that we define as the GAG binding surface [40]. In a similar fashion, on titrating R1, chemical shifts of a selective subset of residues are perturbed, which constitute the receptor binding surface and/or are in the vicinity of the binding surface [53]. For both R1 and heparin titrations, we observed only one set of peaks that correspond to the population average between the free and bound forms in fast exchange on the NMR time scale (figure 1*a*,*b*). On adding R1 to the dp8-bound and dp14-bound monomer complexes, the peaks move along the line joining the M·R1 and M·heparin complexes (figure 1*c*,*d*; electronic supplementary material, figure S1). Considering the new peaks of all residues lie along the straight line between the binary complexes, these must correspond to the population average between the two binary complexes. If a ternary complex were to form, the final peaks will not necessarily lie between the two binary complexes for every single residue, but will be random. Therefore, there is no ternary complex formation and successive R1 additions only result in populating the M·R1 complex.

### 3.2. Binding of CXCR2 to heparin-bound CXCL8 monomer

**Figure 2.**
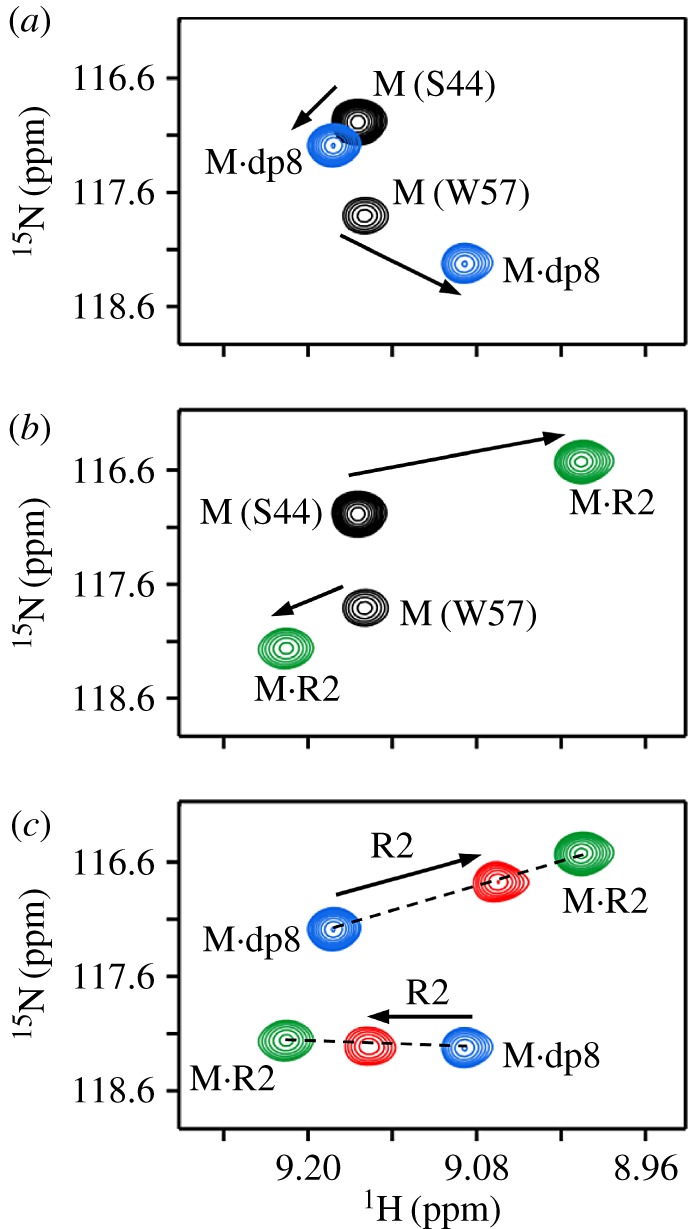
Binding of the CXCR2 N-domain to heparin-bound monomer. Sections of the ^1^H–^15^N HSQC spectrum showing heparin dp8 (*a*) and R2 (*b*) binding to the CXCL8 monomer. The free (M) and final (M·dp8 and M·R1) cross-peaks for residues S44 and W57 are shown in black, blue and green, respectively. (*c*) Section of the same region showing R2 binding to the M·dp8 complex. On successive R2 addition, the new cross-peaks (red) fall on a straight line between the R2-bound and dp8-bound peaks.

Titration profiles for residues S44 and W57 are shown in figure 2. Spectral changes on titrating the CXCR2 N-domain (R2) to monomer were similar to that observed for the R1 titrations (figure 2*b*). As in the case of the R1 titration, on adding R2 to the dp8·monomer complex, the peaks follow the straight line between the M·R2 and M·dp8 complexes, indicating that there is no ternary complex and that successive R2 addition results in populating the M·R2 complex (figure 2*c*; electronic supplementary material, figure S2). The final peaks correspond to the average of the two binary complex populations.

### 3.3. Binding of CXCR1 to heparin-bound CXCL8 dimer

**Figure 3.**
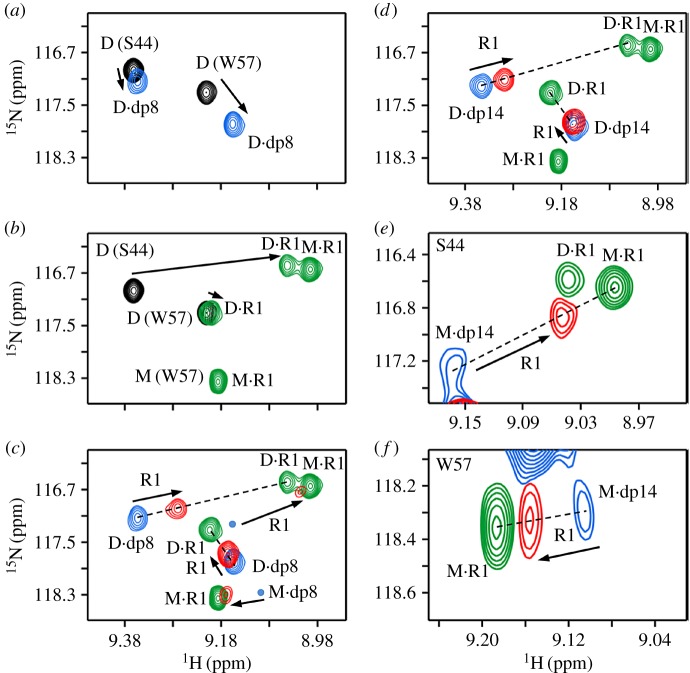
Binding of the CXCR1 N-domain to heparin-bound dimer. (*a*) Section of the ^1^H–^15^N HSQC spectrum showing heparin dp8 binding to the CXCL8 dimer. The initial free (D) and final (D·dp8) cross peaks are shown in black and blue, respectively. (*b*) Section of the same region showing R1 binding to the CXCL8 dimer. The free dimer (D) and R1-bound (M·R1 and D·R1) peaks are shown in black and green, respectively. (*c*) Section of the same region on R1 addition to the D·dp8 complex. The final peaks for the dimer and monomer (red) lie on a straight line joining the dp8-bound and R1-bound peaks indicating no ternary complex. Weakly populated M.dp8 cross peaks (not seen at the contour level shown) are indicated as blue circles. (*d*) Sections of the HSQC spectrum on R1 addition to the D·dp14 complex. Spectra are similar to the dp8 titration. (*e*,*f*) Sections of the same region on R1 addition to the M·dp14 complex. The final peak position (in red) lies on the line joining the two binary complexes.

We first describe the NMR characteristics of the binary complexes of heparin dp8, dp14 and R1 binding to the CXCL8 dimer (D) (figure 3). On titrating dp8 or dp14, the chemical shifts of a selective set of dimer residues, which we define as the heparin-binding surface, are perturbed (figure 3*a*,*d*). On titrating R1, we observed two sets of peaks, which correspond to the R1-bound dimer and R1-bound monomer (figure 3*b*). The free CXCL8 and CXCL8·R1 complexes are in fast exchange, while the M·R1 and D·R1 complexes are in slow exchange. The free dimer is always in equilibrium with the free monomer, and as the monomer binds R1 with much higher affinity, successive titration of R1 populates the M·R1 complex at the expense of the D·R1 complex. The relative populations are dictated by the total protein concentration, and M–D, M–R1 and D–R1 equilibrium constants.

On adding R1 to heparin-bound dimer, the final peaks lie close to the D·heparin peaks (figure 3*c*,*d*). Moreover, for all residues, the new peaks lie in a straight line between the D·R1 and D·heparin complexes, indicating that the peaks are a population average of the two binary complexes, and that there is no ternary complex formation (figure 3*c*,*d*; electronic supplementary material, figure S3). Despite adding approximately 10-fold excess R1, the final peak position lies close to the D·heparin peak, providing compelling evidence that the dimer affinities for dp8 and dp14 are much higher than for R1. In the case of R1 titration to the dp14-bound CXCL8, we also observed peaks corresponding to the monomer (figure 3*e*,*f*). These data also provide convincing evidence that the dp14-bound native monomer is unable to bind the receptor, and demonstrate the power and sensitivity of NMR experiments by allowing simultaneous characterization of whether heparin-bound native dimers and monomers are able to bind the receptor.

**Figure 4.**
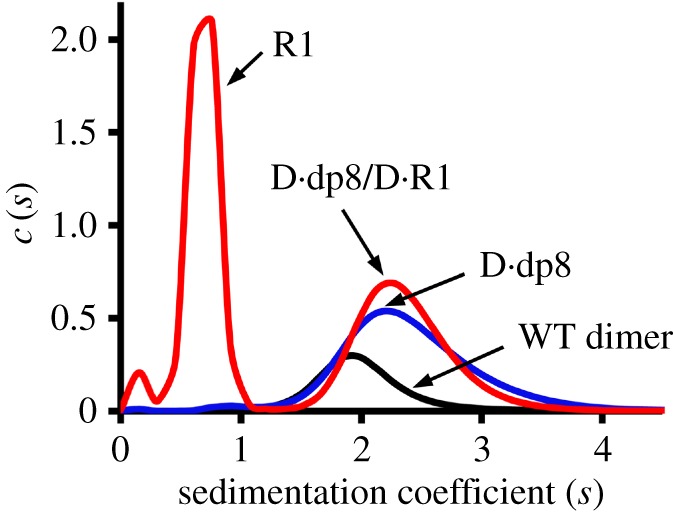
Complex formation from sedimentation velocity. Profiles of the WT CXCL8 dimer (black), the CXCL8·dp8 complex (blue) and the [CXCL8·dp8] + R1 mixture (red). The two peaks observed for the [CXCL8·dp8] + R1 mixture correspond to the free R1 and a population of the CXCL8·dp8 and CXCL8·R1 binary complexes that have similar sedimentation coefficients. For the mixture, the absence of a peak with higher sedimentation coefficient indicates the absence of ternary complex.

We also characterized the binding of R1 to heparin-bound dimer using sedimentation velocity experiments. Sedimentation coefficient (*s*) is proportional to the molecular weight of the complex. We observed that addition of R1 to heparin-bound dimer did not change the *s*-values, providing independent evidence for the absence of ternary complex formation (figure 4).

### 3.4. Binding of CXCR2 to heparin-bound CXCL8 dimer

**Figure 5.**
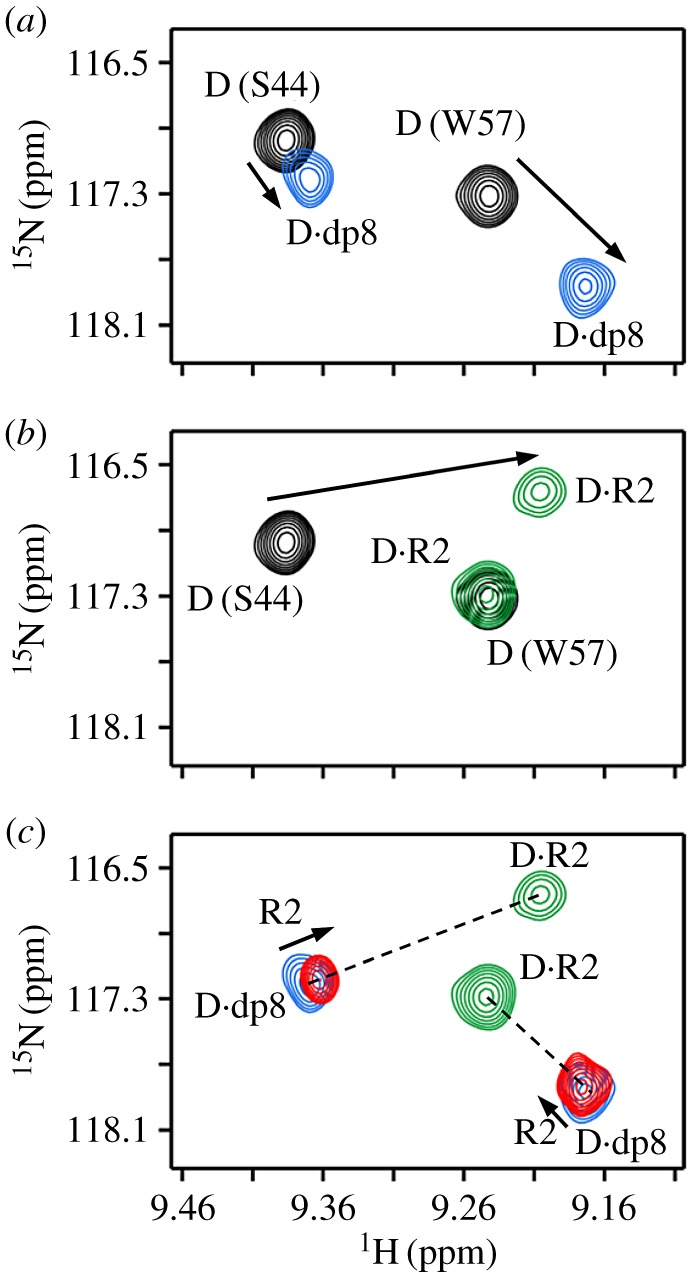
Binding of the CXCR2 N-domain to heparin-bound dimer. Sections of the ^1^H–^15^N HSQC spectrum showing heparin dp8 (*a*) and R2 (*b*) binding to the CXCL8 dimer. The initial free (D) and final (D·dp8 and D·R2) peaks are shown in black, blue and green, respectively. (*c*) Section of the same region on R2 addition to the D·dp8 complex. Excess R2 results in minimal chemical shift change, and moreover, the final peak (red) lies on a straight line between the dp8-bound and R2-bound peaks, indicating no ternary complex formation.

The titration profile of heparin dp8 binding to the CXCL8 dimer is the same as described above (figure 5*a*). Unlike R1, titrating R2 to the CXCL8 dimer results only in one set of peaks corresponding to D·R2 (figure 5*b*). Any M·R2 complex present is negligible. On titrating R2 to the dp8-bound dimer, we observed negligible or no chemical shift changes and the final peaks almost overlapped with the D·dp8 peaks (figure 5*c*; electronic supplementary material, figure S4). Similar to R1, for all residues, the new peaks lie in a straight line between the D·R2 and D·dp8 complexes, indicating that there is no ternary complex and that the dimer binds heparin with much higher affinity than to R2.

### 3.5. Binding of glycosaminoglycan to R1-bound CXCL8 monomer and dimer

Reverse titration of heparin dp8 to R1-bound monomer and dimer also showed no evidence for ternary complexes (electronic supplementary material, figures S5 and S6). In the case of the monomer, similar to what was observed before, the new peaks lie in a straight line between M·R1 and M·dp8 complexes. In the case of the dimer, peaks that correspond to the R1-bound monomer were also observed, which is expected as the monomer binds R1 with higher affinity. On adding dp8, intensity of the R1-bound monomer peaks decreased in intensity and became very weak, while the dimer-bound peaks increased in intensity. This further confirms that the dimer binds heparin with higher affinity, and that heparin binding and dimerization are coupled. Further, the new peaks lie along the line joining the dp8-bound and R1-bound dimer, indicating no ternary complex formation.

### 3.6. Neutrophil receptor activity of heparin-bound CXCL8

**Figure 6.**
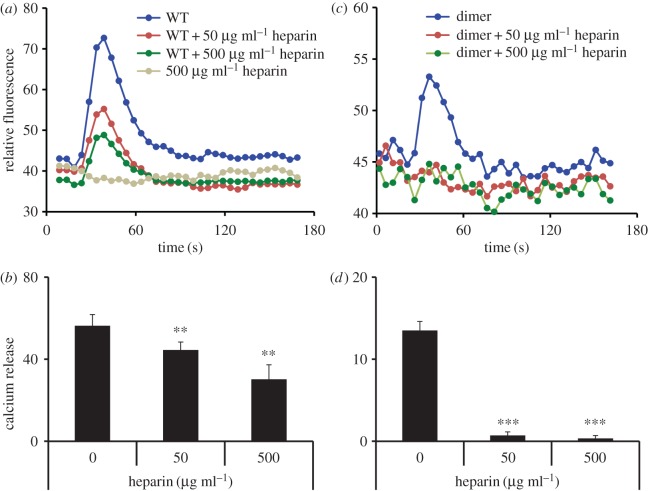
Neutrophil receptor activity. Neutrophil Ca^2+^ release activity of CXCL8 WT and trapped dimer. (*a*,*c*) Traces and (*b*,*d*) histogram plots in the presence of 50 and 500 µg ml^−1^ heparin are shown for WT (*a*,*b*) and trapped dimer (*c*,*d*). Data are representative of four independent donors and are the means ± standard error performed in quadruplicate.

We characterized neutrophil receptor activity of heparin-bound CXCL8 by measuring Ca^2+^ release in human neutrophils. We used CXCL8 WT and trapped dimer, and observe that both show impaired activity in the presence of heparin, and that the loss of activity for trapped dimer was higher compared to the WT (figure 6). Reduced neutrophil Ca^2+^ release activity for heparin-bound CXCL8 has been reported previously [52]. These observations are consistent with the NMR data showing CXCL8 dimer binds heparin with higher affinity, and that there is considerable overlap between receptor-binding and heparin-binding domains.

## 4. Discussion

Chemokine CXCL8 mediates neutrophil recruitment by interacting with GAGs and CXCR1 and CXCR2 receptors [14–20]. CXCL8 exists as monomers and dimers, and animal model studies have shown that GAG interactions and monomer–dimer equilibrium regulate recruitment, and that the recruitment profiles of the monomers and dimers are distinctly different [37,38]. In this study, using solution NMR spectroscopy, we addressed a fundamental question of whether a GAG-bound chemokine can bind the receptors. We used NMR chemical shifts as probes to detect ternary complex formation. Whereas changes in chemical shifts must be interpreted with caution due to their high sensitivity as changes can occur due to small differences in pH and buffer conditions, our observations provide compelling evidence for the lack of ternary complex formation. Our NMR data show that heparin-bound monomer and dimer are unable to bind CXCR1 and CXCR2 receptors, and functional data also show that heparin-bound CXCL8 is impaired for neutrophil receptor activity.

**Figure 7.**
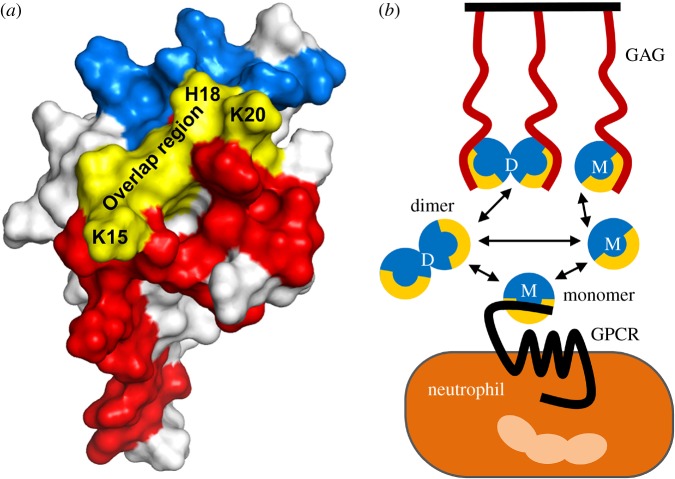
Schematic showing binding of CXCL8 to GAG and receptor. (*a*) Molecular plot of CXCL8 showing CXCR1 N-domain site-I (red and yellow) and heparin GAG (blue and yellow) binding surfaces. The overlap region is shown in yellow. (*b*) Both monomers and dimers bind GAG and the receptor. Dimer is the high-affinity ligand for GAG binding, and monomer is the high-affinity ligand for receptor binding. It is very likely that dimer preferentially exists in the GAG-bound form and the monomer in the free form. Regions that are common to both GAG and receptor binding are shown in yellow. Considering that the receptor binding surface is occluded in the GAG-bound form, only free CXCL8 and not the GAG-bound CXCL8 can bind the receptor.

NMR and functional studies have shown that CXCL8 N-loop I10, T12, Y13, S14, K15, F17, H18, K20 and F21 and adjacent *β*-strand E48 and L49 residues are involved in binding to the CXCR1 N-domain [53,55]. NMR studies also indicate that N-loop I10, T12, Y13, S14, K15 and H18 and *β*-strand E48 and L49 residues mediate binding to the CXCR2 N-domain. NMR and mutational studies have shown that N-loop K15, H18, K20 and K23 and helical residues R60, K64 and R68 mediate binding to heparin [40,50]. These data show that several N-loop residues that are involved in binding to CXCR1 and CXCR2 are also involved in binding heparin. The extent of overlap is schematically shown for CXCR1, and is essentially similar for CXCR2 (figure 7*a*). On the basis of these data and from the observation that the dimer binds GAGs with higher affinity, we propose a model of how GAG interactions regulate receptor function (figure 7*b*).

Whereas we used free heparin for our experiments, several factors come into play as to how chemokines bind *in vivo* GAGs. GAGs, such as HS and CS, are the glycan part of the PGs that exist in different forms. PGs span the lipid bilayer in the endothelium and the epithelium, and GAGs are covalently attached to the ectodomain. PGs are secreted and are part of the ECM where they exist as large macromolecular complexes with matrix proteins [24], and are part of the glycocalyx that dominates the luminal side of the endothelium [32]. PG ectodomains are also shed at different locations due to cleavage by proteases [35]. Therefore, *in vivo* binding is critically dependent on the local environment, and accessibility of GAG chains within and between PGs due to proximity and geometric constraints. Further, the dimensions of the glycocalyx are much larger (greater than 500 nm thick) compared to a chemokine (approx. 3 nm) or a typical GAG (approx. 10 to 30 nm). Considering our simple *in vitro* experimental conditions show no evidence for binding of heparin-bound CXCL8 to receptor N-domain, it is unlikely that GAG-bound CXCL8 can bind the intact receptor on neutrophils under *in vivo* conditions that are more complex, and involves chemokines binding to much longer GAGs and restricted accessibility due to steric factors and the crowded environment.

We propose that only the free chemokine, and by extension chemotactic gradients, drive neutrophil trafficking from circulation to the tissue. Our data and conclusions do not rule out a role for haptotactic gradients, as haptotactic gradients determine the makeup of the chemotactic gradients. This is because the GAG-bound chemokine dictates the levels of free soluble chemokine. We propose that any chemokine that is washed away with flow is likely to be negligible compared to the large reservoir of GAG-bound chemokines. It is also possible that chemokines are not as quickly dissipated with flow due to local steric constraints, and that their lifetime is long enough to be able to bind the receptor directing neutrophils to their target site. Further, any chemokine that is lost is also continuously replenished by chemokines from the ECM. Previous studies for the related NACs CXCL1 and CXCL5 also indicate that it is unlikely that the heparin-bound chemokine can bind the receptor [41,42]. At this time, it is believed for most chemokines that haptotactic gradients are the underlying mechanism driving leucocyte trafficking. However, direct experimental proof for ternary complex formation or that GAG-bound chemokine alone activates the receptor is lacking, and experimental studies similar to those reported here are essential to establish the structural basis underlying chemokine gradient formation, receptor activation and leucocyte trafficking.

## Supplementary Material

Supplementary NMR HSQC titiration figures 1 to 6

## Data accessibility

Data deposition is not applicable to this paper.
