# Supplementary material for: Heparin-bound chemokine CXCL8 monomer and dimer are impaired for CXCR1 and CXCR2 activation: implications for gradients and neutrophil trafficking
Source: Open Biol. 2017 Nov 8;7(11):170168. doi: 10.1098/rsob.170168 (PMC5717344; doi:10.1098/rsob.170168)
Supplement: Supplementary NMR HSQC titiration figures 1 to 6 [file rsob170168supp1.pdf]

Open Biology
Supplementary Material

Heparin-bound chemokine CXCL8 monomer and dimer are impaired for CXCR1 and CXCR2 activation: implications for gradients and neutrophil trafficking

Prem Raj B. Joseph[1,2] , Kirti V. Sawant[1,2] and Krishna Rajarathnam[1,2,3 *]

[1]Department of Biochemistry and Molecular Biology, University of Texas Medical Branch, Galveston, TX, USA.
[2]Sealy Center for Structural Biology and Molecular Biophysics, University of Texas Medical Branch, Galveston, TX, USA.
[3]Department of Microbiology and Immunology, University of Texas Medical Branch, Galveston, TX, USA.

***Author for Correspondence:**
Dr. Krishna Rajarathnam
krrajara@utmb.edu

doi: 10.1098/rsob.170168

Supplementary Figures

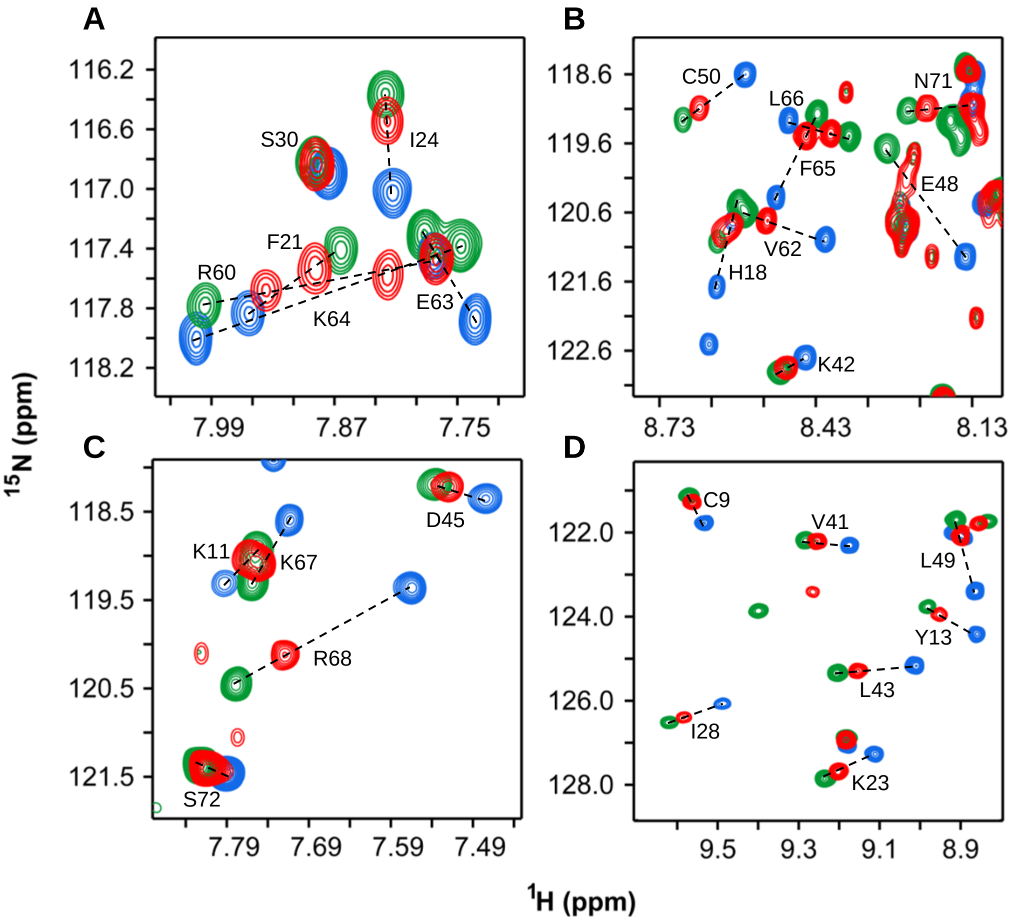

Supplementary Figure 1. Binding of CXCR1 N-domain to heparin-bound monomer. **(A-D)** Different sections of the ^{1}H-^{15}N HSQC spectrum showing binding profiles on R1 addition to M•dp8 complex. Dotted lines are drawn between the M•dp8 (blue) and M•R1 (green) complexes. The cross peaks that correspond to [M•dp8]+R1 (red) lie on this straight line.

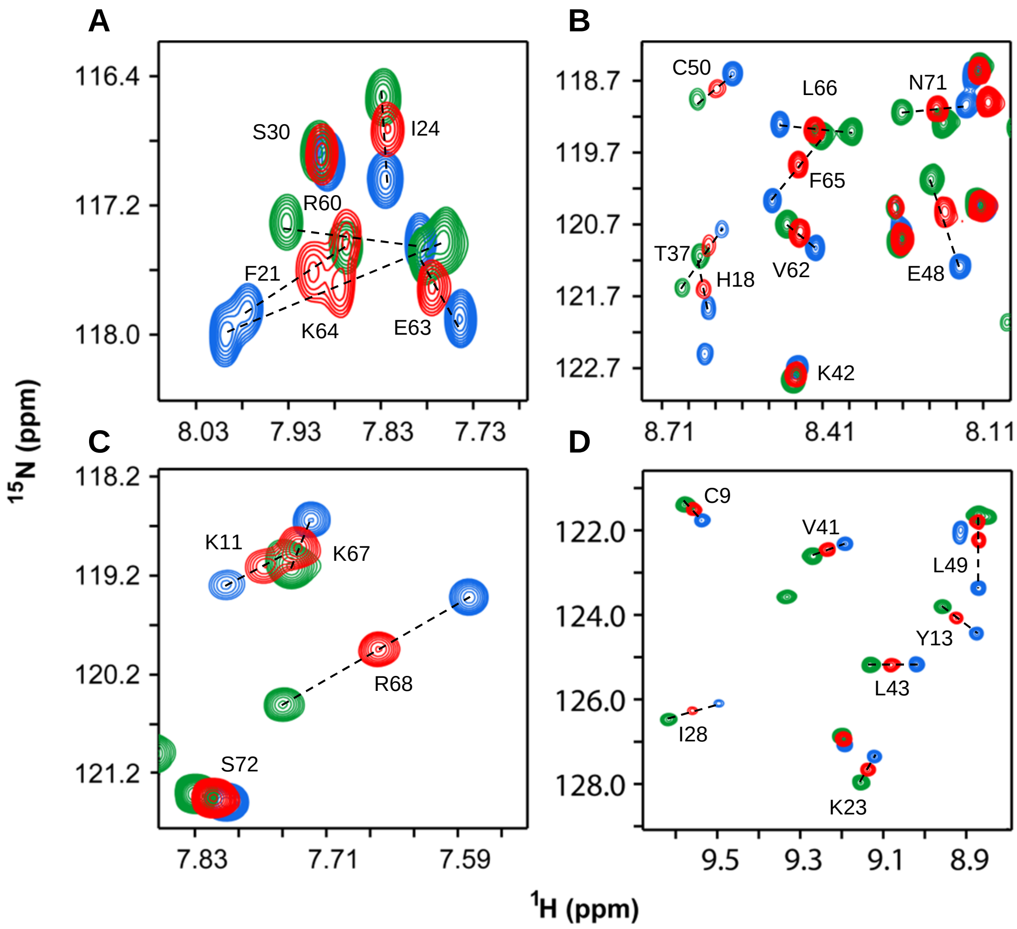

Supplementary Figure 2. Binding of CXCR2 N-domain to heparin-bound monomer. **(A-D)** Different sections of the ^{1}H-^{15}N HSQC spectrum showing binding profiles on R2 addition to M•dp8 complex. Dotted lines are drawn between the M•dp8 (blue) and M•R2 (green) complexes. The cross peaks corresponding to [M•dp8]+R2 (red) lie on this straight line.

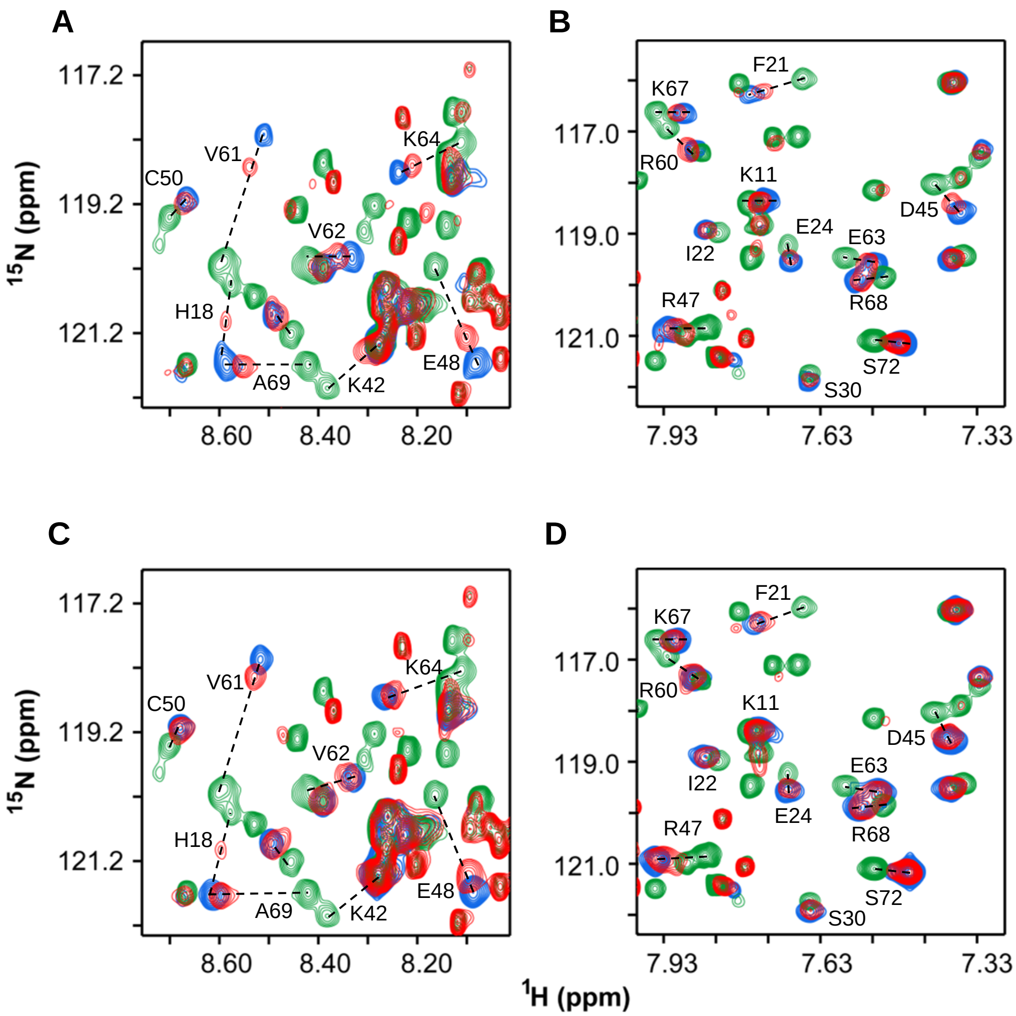

Supplementary Figure 3. Binding of CXCR1 N-domain to heparin-bound dimer. Different sections of the ^{1}H-^{15}N HSQC spectrum showing binding profiles of R1 addition to D•dp8 (panels **A** and **B**) and D•dp14 complexes (panels **C** and **D**). Dotted lines are drawn between the heparin-bound (blue) and R1-bound (green) complexes. For every residue, the cross peak (red) corresponding to [D•dp8]+R1 (panels **A** and **B**) or [D•dp14]+R1 (panels **C** and **D**) lies on the straight line joining the heparin-bound and R1-bound complexes.

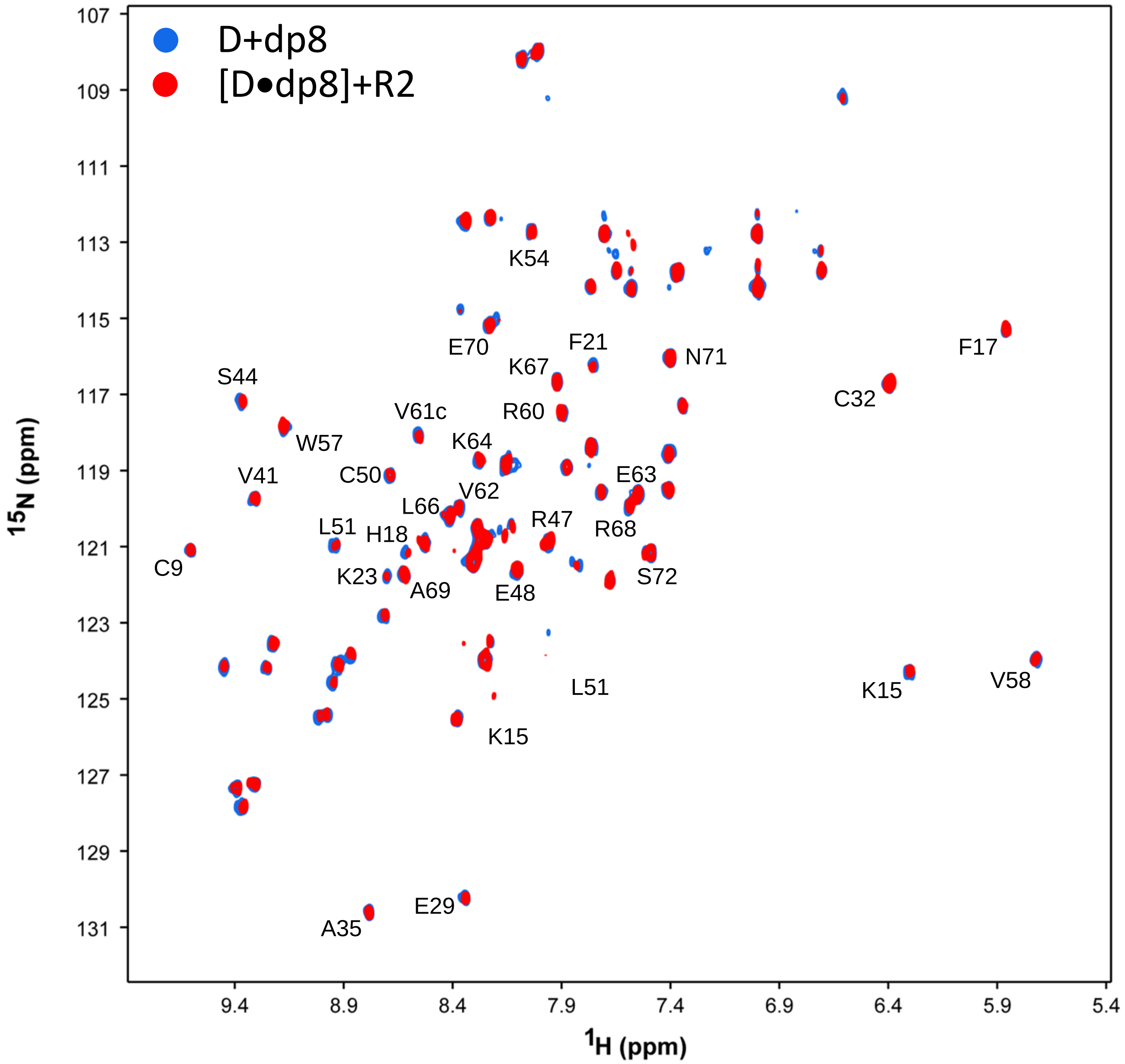

Supplementary Figure 4. Binding of CXCR2 N-domain to heparin-bound dimer. ^{1}H-^{15}N HSQC spectrum showing binding profiles on R2 addition to D•dp8 complex. Titration of R2 results in negligible chemical shift change, indicating that most of the protein exists in the GAG-bound form. The spectrum of the D.R2 is not shown for clarity. Further, in all cases, the new peaks (red) lie in a straight line between D•dp8 (blue) and D•R2 (not shown) complexes.

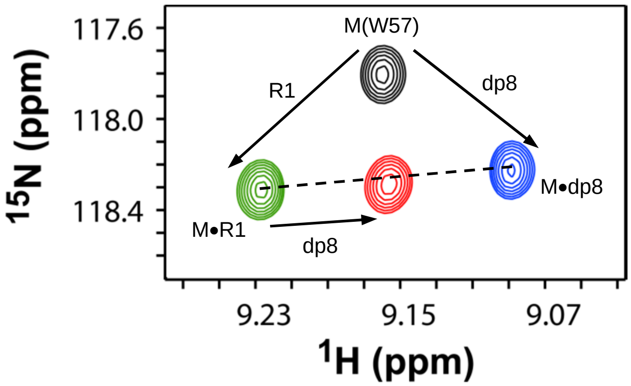

Supplementary Figure 5. Binding of heparin to CXCR1 N-domain-bound monomer. A section of the ^{1}H-^{15}N HSQC spectrum showing binding profile on dp8 addition to M•R1 complex. Dotted lines are drawn between the M•R1 (green) and M•dp8 (blue) complexes. The cross peaks corresponding to [M•dp8]+R1 (red) lie on this straight line.

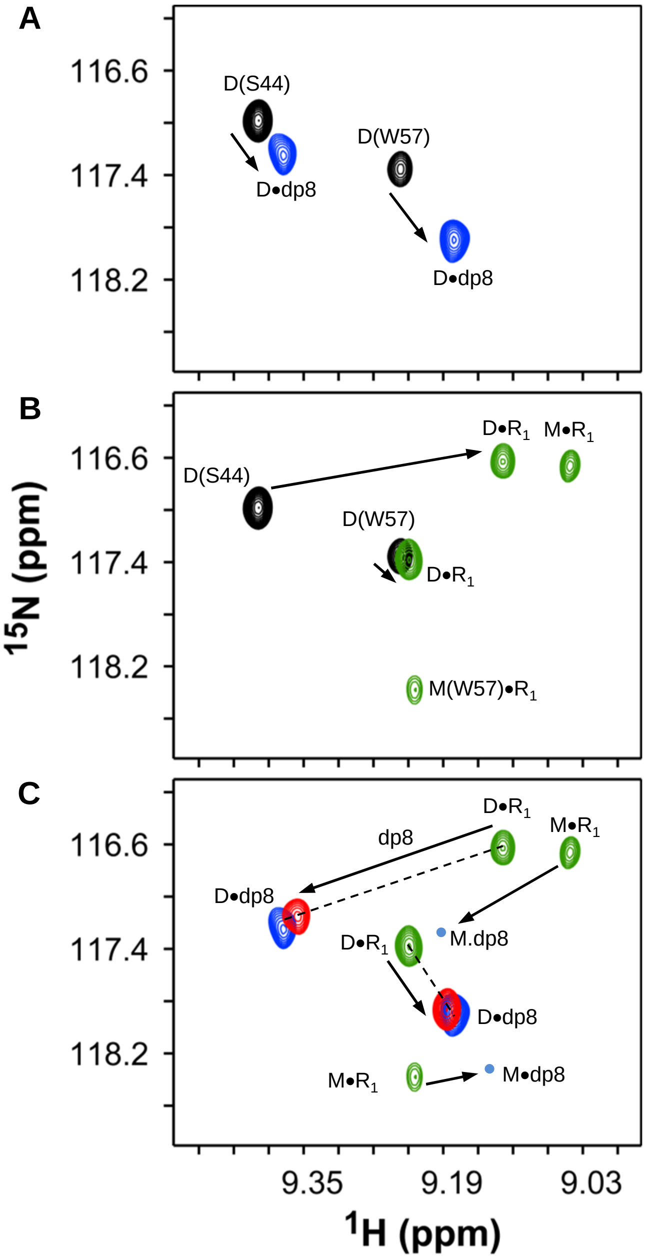

Supplementary Figure 6. Binding of heparin to CXCR1 N-domain-bound dimer. **(A)** Section of the ^{1}H-^{15}N HSQC spectrum showing heparin dp8 binding to CXCL8 dimer. The initial free (D) and final (D•GAG) cross peaks are shown in black and blue, respectively. **(B)** Section of the same region showing R1 binding to CXCL8 dimer. The free dimer (D) and bound monomer (M•R1) and dimer (D•R1) peaks are shown in black and green, respectively. **(C)** Section of the same region on dp8 addition to D•R1 complex. The final peaks (red) lie on a straight line between the dp8-bound and R1-bound peaks indicating no ternary complex formation. The lowly populated M.dp8 cross peaks (not seen at the contour level shown) are indicated as blue circles.
